# Applications of art therapy/occupational therapy in neuropsychic disorders: raw art objectives: stimulation, creativity, catharsis, socialization

**DOI:** 10.1192/j.eurpsy.2024.1083

**Published:** 2024-08-27

**Authors:** E. Chirila

**Affiliations:** Centrul comunitar judeţean, Complex de servicii sociale comunitare pentru copii și adulţi, Direcției Generale de Asistență Socială și Protecția Copilului Cluj, Cluj-Napoca, Romania

## Abstract

**Introduction:**

Art therapy, occupational and play therapy support the idea that man becomes what he is through activity. We accept man in his totality, we oppose the rigid boundaries between “sick” and “healthy”, there is no contradiction between these states, but only an unbroken chain of neighboring nuances, of gradual differences of human life.

**Objectives:**

Therapies based on the visual arts are also used in the discharge of repressed experiences, called in psychoanalytic terms “catharsis”, with the aim of preventing or remedying dysfunctions, facilitating maximum adaptability of the beneficiary, regardless of the degree of handicap. The activities of occupational therapy and ergotherapy has and productive objectives, in accordance with the outstanding, hidden skills, correlated with the current demands on the labor market. Art forms called: “Raw Art” can be practiced.

**Methods:**

Traditional crafts and visual arts are reinvented as therapeutic methods. We use pottery and sculptural artistic ceramics with elements from the history of archaic arts connected with contemporary visual arts (pictures, sculpture, graphics, mixed arts and multimedia)

**Figure:**
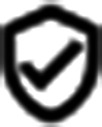

**Results:**

The creative process and symbolic communication, associated with narration and imitation, develop new ways of communication, new ways of self-expression, new ways of seeing things. Throughout this process, people are more productive, more efficient, focused, calm and self-satisfied. (2014- Emilia Chirilă The role of art therapy in self-knowledge, self-esteem and interpersonal relationships in children with emotional disorders”- THE SECOND WORLD CONGRESS ON RESILIENCE: FROM PERSON TO SOCIETY may 8-10 Timisoara Romania 1139-1145editors: M Tomita, S.Cace – MEDIUMOND- INTERNATIONAL PROCEEDINGS copyright 2014 by MEDIMOND srl 40065 Piamond (Bologna) Italy -Printed May 2014 by Editografica.Bologna Italy - ISBN 978-88-7587)

**Image:**

**Figure:**
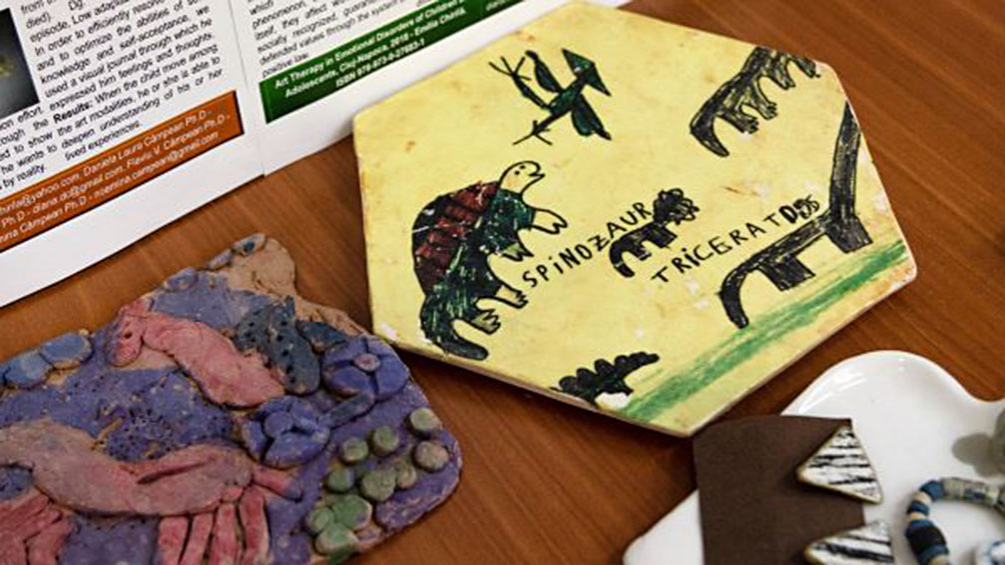

**Image 2:**

**Figure:**
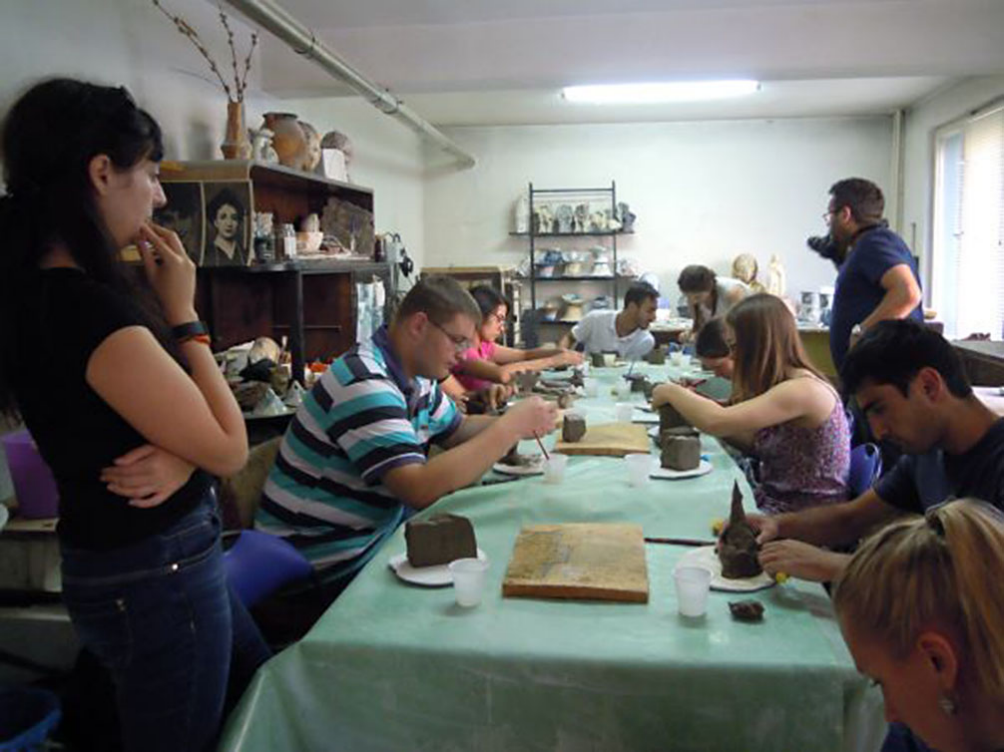

**Image 3:**

**Figure:**
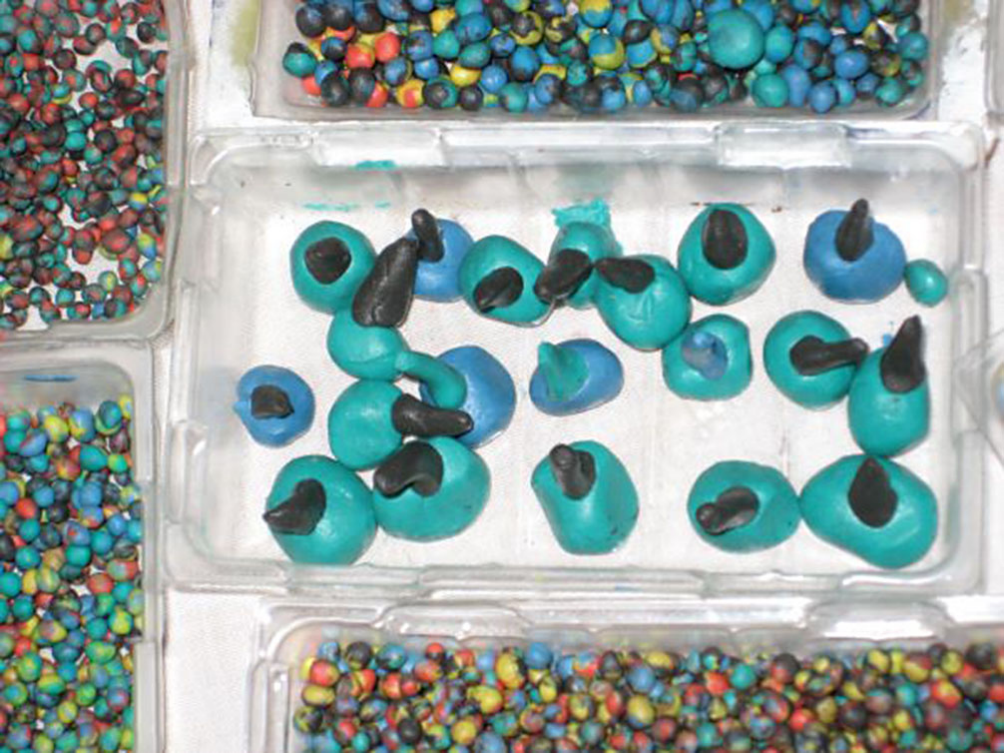

**Conclusions:**

People who do not have a sufficiently developed vocabulary to be able to express themselves feel very good practicing art as a natural means of communication. Discovering the skills of the beneficiary, through the artistic product and the awareness of emotions and feelings open up ways to a more effective non-verbal communication.

**Disclosure of Interest:**

None Declared

